# Supplementary material for: Need for high-resolution Genetic Analysis in iPSC: Results and Lessons from the ForIPS Consortium
Source: Sci Rep. 2018 Nov 21;8:17201. doi: 10.1038/s41598-018-35506-0 (PMC6249203; doi:10.1038/s41598-018-35506-0)
Supplement: Supplementary file 1 — Supplementary information [file 41598_2018_35506_MOESM1_ESM.pdf]

Supplementary Information:

Need for high-resolution Genetic Analysis in iPSC: Results and Lessons from the ForIPS Consortium

Authors

Bernt Popp[1, 6], Mandy Krumbiegel[1, 6], Janina Grosch[2], Annika Sommer[3], Steffen Uebe[1], Zacharias Kohl[2], Sonja Plötz[2], Michaela Farrell[3], Udo Trautmann[1], Cornelia Kraus[1], Arif B. Ekici[1], Reza Asadollahi[5], Martin Regensburger[3], Katharina Günther[4], Anita Rauch[5], Frank Edenhofer[4], Jürgen Winkler[2], Beate Winner[3], André Reis[1, *]

Affiliations

[1]Institute of Human Genetics, University Hospital Erlangen, Friedrich-Alexander-Universität Erlangen-Nürnberg (FAU), Schwabachanlage 10, 91054 Erlangen, Germany

[2]Department of Molecular Neurology, University Hospital Erlangen, Friedrich-Alexander-Universität Erlangen-Nürnberg (FAU), Schwabachanlage 6, Erlangen, Germany

[3]Department of Stem Cell Biology, University Hospital Erlangen, Friedrich-Alexander-Universität Erlangen-Nürnberg (FAU), Glückstrasse 6, Erlangen, Germany

[4]Stem Cell Biology and Regenerative Medicine Group, Institute of Anatomy and Cell Biology, Julius-Maximilians-University of Würzburg, Würzburg, Germany

[5]Institute of Medical Genetics, University of Zurich, Schlieren-Zurich, Switzerland.

[6]Co-first author

[*]Corresponding author

andre.reis@uk-erlangen.de (A.Re.)

Competing Interests

The authors declare no competing interests.

Keywords

iPSC; ForIPS; genetic quality control; exome sequencing; chromosomal microarray

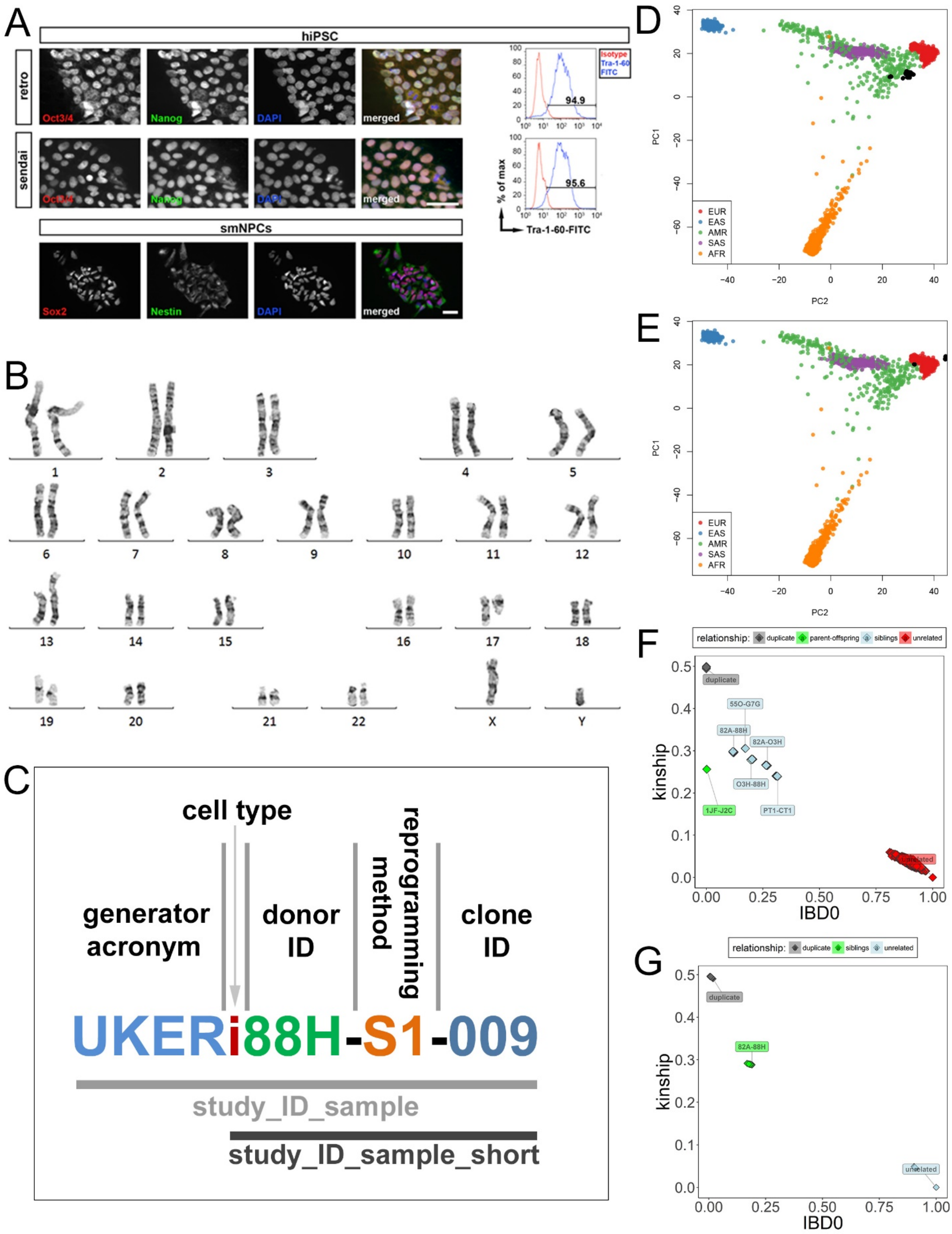

Figure S1 **(A)** Example of pluripotency confirmation by positive staining for POU5F1 (Oct3/4) and NANOG (Nanog), and fluorescence-activated cell scanning (FACS) analysis for TRA-1-60 (Tra-1-160) in the iPSC lines “i88H-R1-001”and “i88H-S1-002”. **(B)** Example of conventional karyotyping in a iPSC line (P_44486) excluded because of a large structural chromosomal rearrangement involving chromosomes 13 and 17 (46,XY,t(13;17)(p10;p10)). **(C)** Schematic explanation of the nomenclature encoding

used for cell lines in the ForIPS consortium which is based on the hPSCreg[1] recommendations. **(D)** Individual variant data from CMA projected onto the first two principle components from the 1000genomes project (genome data; colors represent the different populations included in the 1000genomes study[2]) using the akt-kit[3] confirms European or admixed European (black dots) of the cohort as reported. **(E)** Same analysis as in (D) for samples with exome data using the 1000genomes exome data. Note the slight differences which are caused by missing variants between the precomputed data from 1000genomes and the variants from CMA or exomes. **(F)** Plot of IBD0 and kinship coefficient calculated using the akt-kit from CMA data recapitulates the reported kinship relationships in the cohort. **(G)** Same analysis as in (F) for samples with exome data.

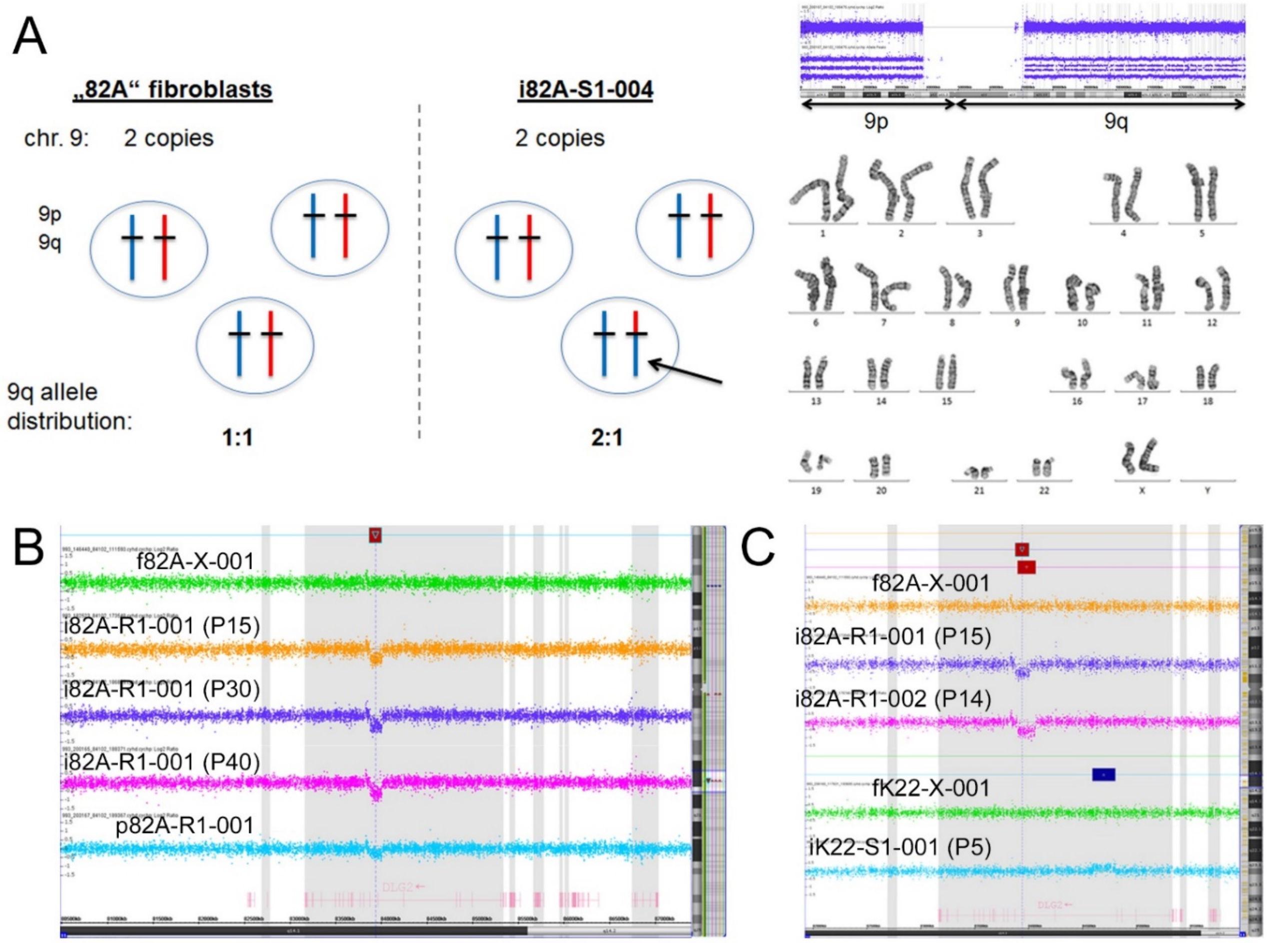

<u>Figure S2</u> (A) Copy number analysis (right top) as well as conventional karyotyping (right bottom) revealed unremarkable results on chromosome 9 of one Sendai clone, but allele peak distribution uncovered a copy neutral allelic imbalance on the long arm of chromosome 9 in the Sendai clone i82A-S1-004, indicating a 2:1 distribution of parental alleles (left). **(B)** The somatic 11q14.1 deletion affecting the *DLG2* gene in the “p82A-R1-001” NPC derived from the RiPSC clone (“i82A-R1-001”) seems to underlie negative selection and occurs only in ~50 % of the NPCs, while it is present at comparable frequencies in the higher passage RiPSCs. **(C)** Example of a putative hotspot region at the *DLG2* gene locus. Overlapping deletions were identified in the two RiPSCs “i82A-R1-001” and “i82A-R1-002” from individual 82A and a non-overlapping deletion in the independent SiPSC “iK22-S1-001” from individual K22.

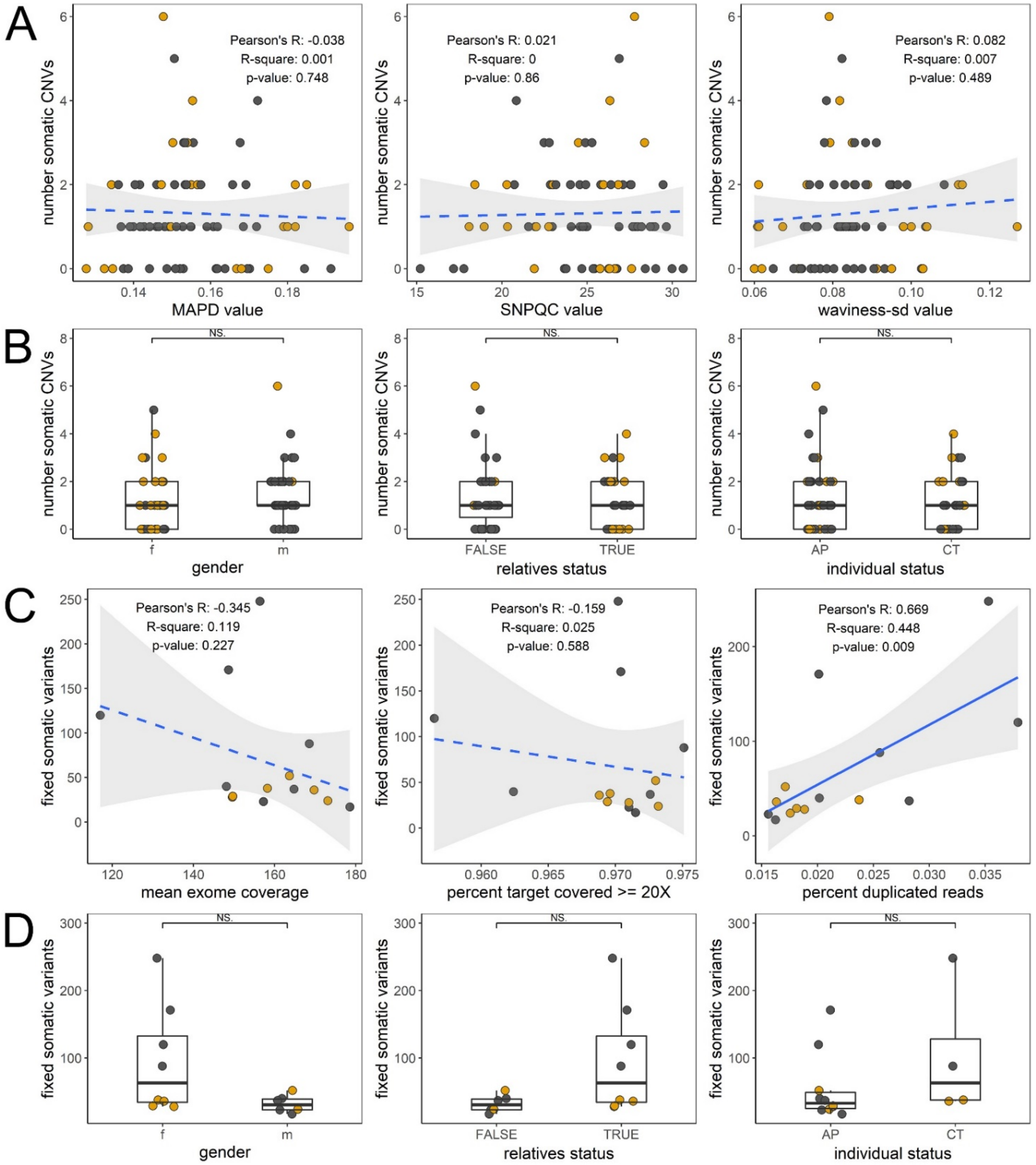

Figure S3 Analysis of possible confounders. **(A)** No correlation of somatic CNV number with the CMA quality measures MAPD, SNPQC, waviness-sd was identified. **(B)** No significant differences in somatic CNV count was identified when data was grouped by gender (f = female, m = male), relatedness (TRUE = related to other individuals from cohort, FALSE = unrelated to other individuals from cohort) and affected status (AP = affected person, CT = control). **(C)** No correlation of fixed somatic SNV/indel number with the exome quality measures mean read coverage

and percent target covered >= 20X was identified. Fixed somatic SNV/indel number correlated with duplication rate, however all samples have a low PCR duplication rate, this is likely a low duplication rate (0.05) and the correlation is strongly influenced by two outlier samples which makes it likely that this is a spurious correlation. **(D)** No significant differences in fixed somatic SNV/indel count was identified when data was grouped by gender, relatedness and affected status.

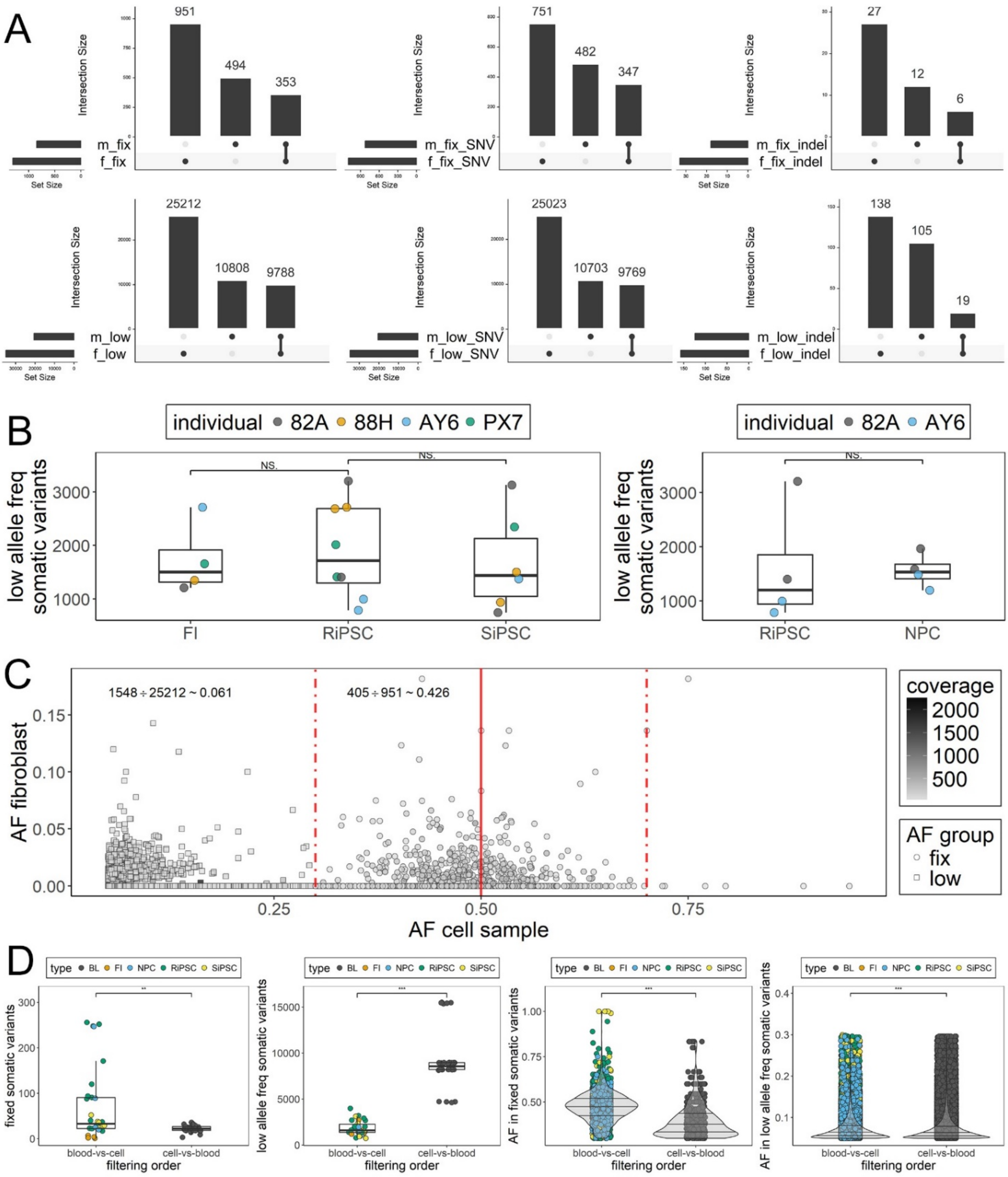

Figure S4 **(A)** UpSet plots comparing the freebayes (f;[4]) vs. the MuTect2 (m;[5]) call-sets for fixed and low allele frequency variants and split by variant type (SNV and indel). MuTect2 generally calls less variants then the freebayes calling/filtering. While the overlap for the SNVs is good, the overlap between the indel call-sets is relatively low, despite normalization and left-alignment using bcftools.[6] **(B)** Comparison of the total number of low frequency somatic SNVs/indels in independently reprogrammed

SiPSC and RiPSCs from four fibroblast (FI) donors (left) and in RiPSC and derived NPCs /right). No significant differences were detected (two sided Wilcoxon signed-rank test). Like in the fixed variant analysis, certain cultures have a higher variant load. **(C)** Dot-plot showing the distribution of allele fraction (AF) in the analyzed iPSC cell cultures (x-axis) and their corresponding fibroblast culture (y-axis) with each point representing a variant shaded by read coverage in the iPSC exome (bright = low, dark = high read coverage at the respective variant position). Dotted vertical lines mark the expected AF for a heterozygous fixed variant (0.5) and typical variabilities seen in short read sequencing (0.3 to 0.7). Variants below the 0.3 AF fraction and categorized as low AF have a much lower probability to have evidence in fibroblasts then the fixed variants (0.061 vs 0.426). This indicates that these low AF variants have not been propagated to the iPSC lines. **(D)** To further analyze whether the low AF variants identified in cell cultures are predominantly real or artefacts, we inverted the freebayes filtering step to search for variants with evidence in the blood sample but not in each cell line from that individual ("cell-vs-blood" analysis, as opposed to the usual "blood-vs-cell" analysis). The hypothesis was that low AF variants would be artefacts if no significant difference was found between these two analyses. In fact, the results plotted as box- and dot-plots show highly significant differences in both fixed and low SNV/indel count and in AF distribution between the groups (two sided Wilcoxon signed-rank test). The observation that blood has much less fixed- but a lot more low-variants also indicates that the respective cell-pools the blood DNA-samples has been derived was larger than for the cultured cells. Also, this analysis supports our model of random genetic drift induced by cell picking as source of the fixed-variants in iPSCs.

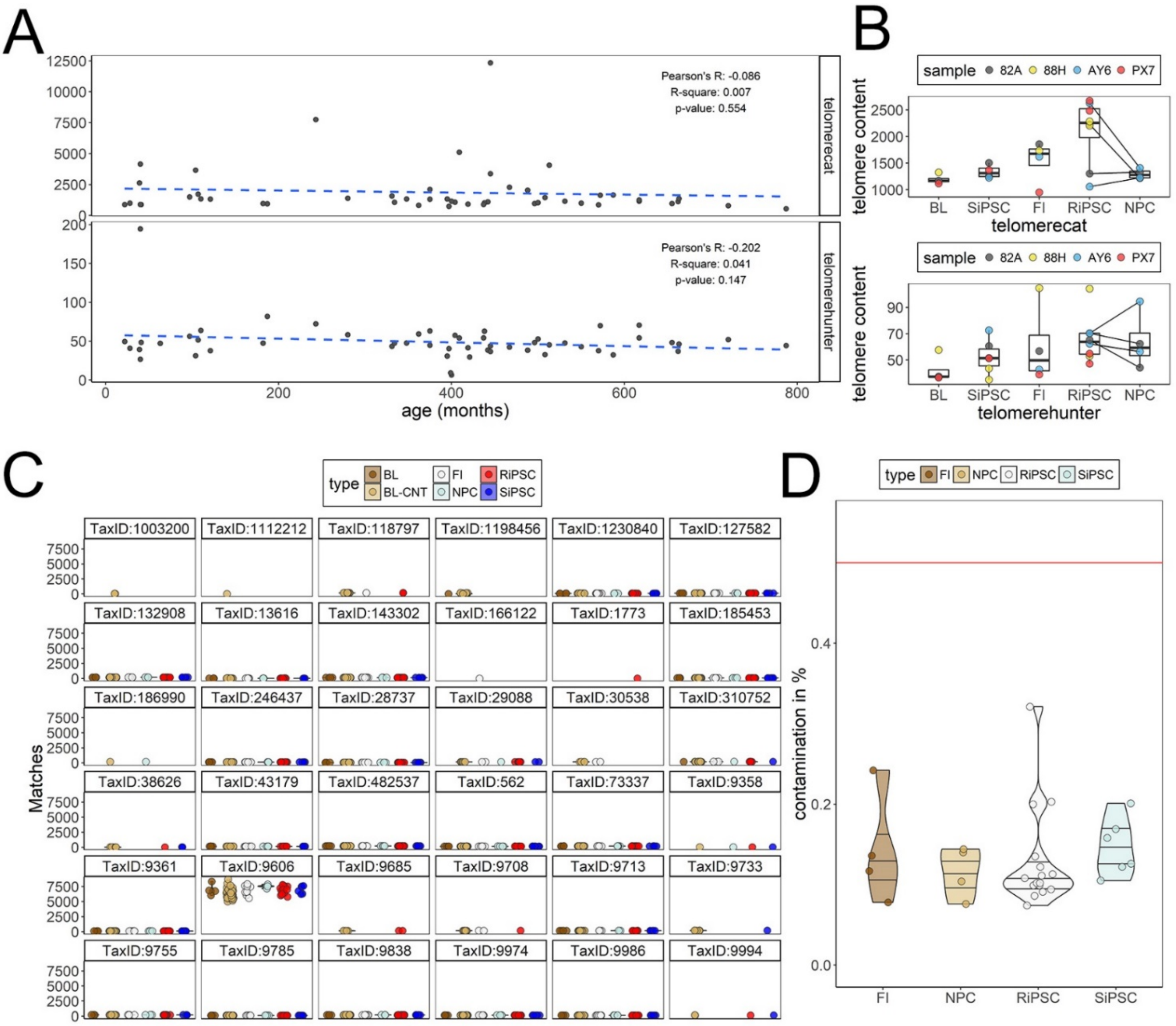

<u>Figure S5</u> **(A)** Telomere content of all 53 in-house exome controls samples estimated from off-target telomeric reads by two different algorithms, telomerecat (upper panel;[7]) and telomerehunter (lower panel;[8]) plotted vs. the individuals' age in months. No significant correlation of telomere content with higher age was identified in this control cohort. **(B)** Box- and dot-plot of the telomere content estimated by telomerecat (upper panel) and telomerehunter (lower panel) grouped by cell type (BL = blood, FI = fibroblast; RiPSCs and thereof derived NPCs are connected by lines). Differences between blood and cultured cells are evident but results differ between algorithms. **(C)** Results from the microorganism contamination analysis using the BBSketch MinHash algorithm, with k-mer matches plotted for each sample grouped by cell type and faceted by organism TaxID. Matches are comparable for all organisms and all samples are confidently assigned as human (TaxID: 9606) without evidence for significant contamination. **(D)** Violin- and dot-plot for the cross-sample

contamination in the exome files grouped by cell type as estimated by ContEst[9]. All samples are below the recommended 0.5% cut-off (red line), which indicates no significant contamination with other human DNA.

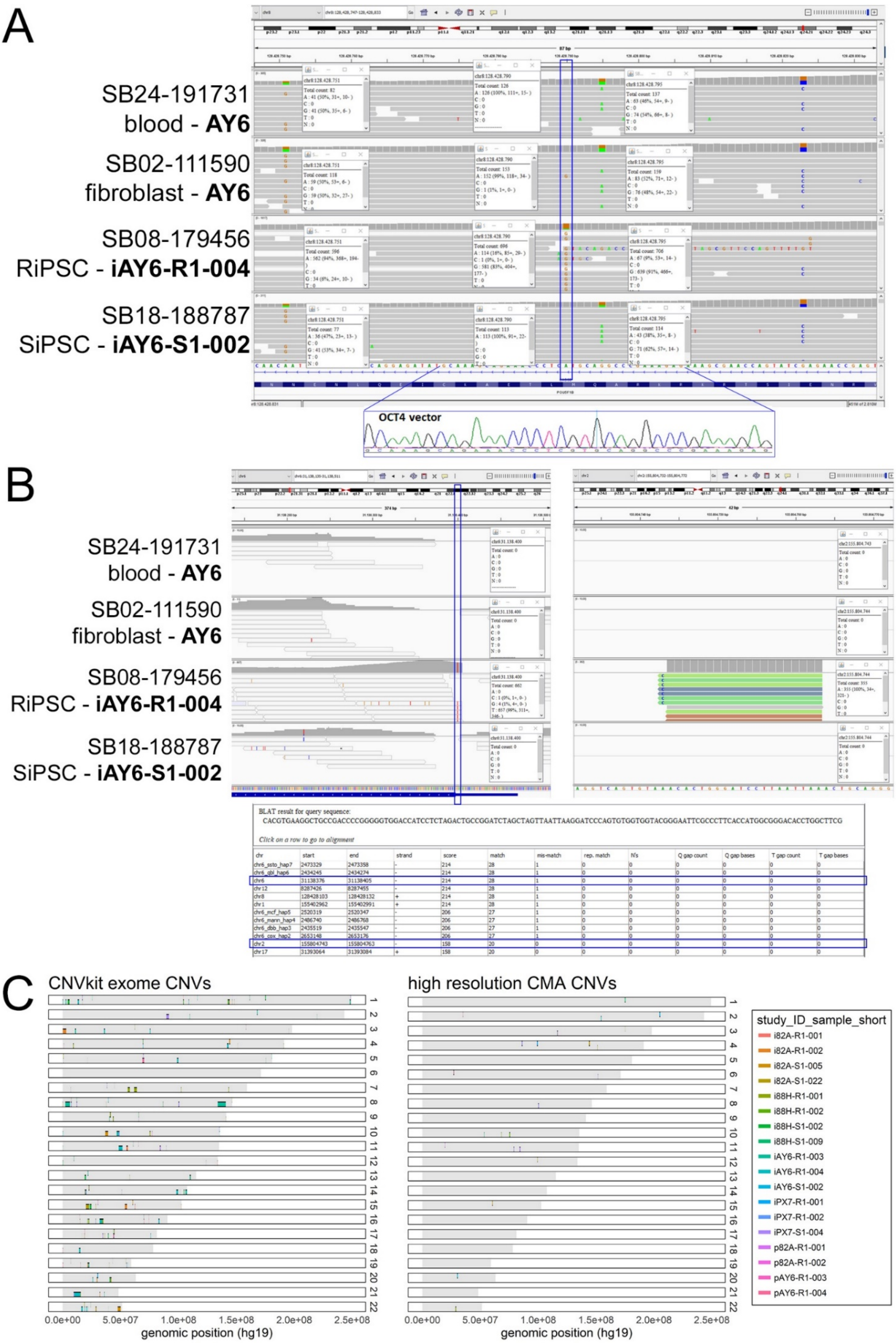

Figure S6 **(A)** IGV snapshot for the blood and fibroblast and an RiPSC ("iAY6-R1-004") and SiPSC ("iAY6-S1-002") sample from individual AY6 at the *POU5F1B* gene-

locus (chr8[hg19]:128428747-128428833; homologous to *POU5F1*) shows a different coverage and SNV profile for the RiPSC sample. The high allele frequency of 581/696 reads for the A>G substitution at position chr8:128428790 (blue highlight) and lacking read evidence in the other samples indicates the insertion of the *POU5F1* (OCT4) vector carrying this substitution. Together with the reduction of the AF for the other variant positions at this locus (chr8[hg19]:128428751A>G, chr8[hg19]:128428795A>G) these results indicate about 3-4 insertions of the vector in the genome. The Sanger trace shows the corresponding nucleotide sequence of the vector used with the respective base-exchanges. **(B)** IGV snapshot for the same samples as in (A) at the *POU5F1* gene-locus (chr6[hg19]:31138135-31138511) showing an abnormal profile at the beginning of exon 1 with higher coverage and a base-exchange not present in the other samples (blue highlight) for the RiPSC sample (left panel). BLAT analysis of split-reads and discordantly mapped read-pairs and identified an insertion breakpoint on chromosome (right panel) **(C)** Comparison of the CNV distribution between CMA and exome analysis. Duplications are in the upper half and deletions in the lower half of each chromosome. CNV calls are colored by sample. Note that there are more calls from the exome data and that these are generally larger for deletions, indicating that exome CNV calls might be noisier and may have a higher false-positive rate. However, in in contrast to CMA data the exome CNVs have not been manually curated after calling.

File S1 (*sample-overview*) This Excel file contains 4 worksheets. A "*summary*" detailing the information for each sheet and all data-columns, "*study_individuals*" with detailed description of all individuals in this study, "*study_samples*" with detailed description of all samples analyzed in this study, "*in-house-Exome_controls*" with detailed description of all samples used as in-house exome controls for this study and "*DistributedLines*" with detailed descriptions of all iPSC lines and whether they have been distributed to subprojects for functional studies at the time of the final project report or whether they could be recommended for distribution considering the results of different genetic QC steps.

File S2 (*QC*) This Excel file contains 7 worksheets. A "*summary*" detailing the information for each sheet and all data-columns, "*Exome-coverage_stats*" with detailed coverage statistics of the exome runs from all 36 samples and 53 in-house controls sequenced on the same 3 machine runs, "*Array_QC*" with detailed quality control statistics of the Array runs from all 108 samples, "*Exome_identity*" with pairwise identity and kinship calculations for all samples with exome sequencing, "*Array_identity*" with pairwise identity and kinship calculations for all samples with Array analysis, "*fingerprinting*" with results of genetic fingerprinting analysis performed to ensure sample identity and "*karyotyping*" with results of conventional karyotyping analysis performed as QC first step for all samples analyzed at the Institute of Human Genetics, Friedrich-Alexander-Universität Erlangen-Nürnberg (FAU).

File S3 (*allCNVs*) This Excel file contains 7 worksheets. A "*summary*" detailing the information for each sheet and all data-columns, "stats_Array_allCNVs" with count statistics for array CNVs, "*stats_CNVkit_exome-aberrations*" with count statistics for exome CNVs, "*Array_allCNVs*" with a summary of germline and somatic CNVs detected in all samples using Chromosome Analysis Suite (ChAS), "*Array_refFlatgenes*" with a list of array CNVs split by affected genes to allow comparison with genesets, "*CNVkit_exome-aberrations*" with CNV calls from exome data using CNVkit 0.9.2a0 and "*CNVkit_refFlatgenes*" with a list of exome CNVs split by affected genes to allow comparison with genesets.

File S4 (*allSomaticSNVs*) This Excel file contains 5 worksheets. A "*summary*" detailing the information for each sheet and all data-columns, "stats_freebayes" with count statistics for somatic variant calls using freebayes v1.1.0-dirty, "stats_mutect2"

with count statistics for somatic variant calls using mutect2 from GATK 3.7-0-gcfedb67, “freebayes_somatic“ with somatic variant calls using freebayes v1.1.0-dirty and “mutect2_somatic” with somatic variant calls using mutect2 from GATK 3.7-0-gcfedb67.

File S5 (*mitochondria-telomeres-contamination*) This Excel file contains 6 worksheets. A “*summary*” detailing the information for each sheet and all data-columns, “*mitochondria*” with results from analysis of mitochondrial genome dosis, “*telomerehunter*” with results from analysis of telomere content by Telomerehunter v1.0.4, “*telomerecat*” with results from analysis of telomere content by telomerecat-3.1.2, “*sendsketch*” with results from contamination analysis for microrganisms using sendsketch from bbmap 37.78 and “*ContEst*” with results from population-based contamination detection by GATKs ContEst.

File S6 (*genesets*) This Excel file contains 5 worksheets. A “*summary*” detailing the information for each sheet and all data-columns, “CGC_20171121” with data from "Census_allTue Nov 21 18_44_41 2017.csv" downloaded on 2017-11-21 from http://cancer.sanger.ac.uk/census, “OMIMmorbidmap-key3_20171122” with data from "morbidmap.txt" downloaded on 2017-11-22 from "https://data.omim.org/downloads/kL9ymp0bQTCeijcWVwOmdQ/morbidmap.txt" filtered for entries with "Phenotype mapping key" value = 3 and “HPA-1437elevatedbrain_20171122” with data from "tissue_specificity_rna_cerebral.tsv" for 1437 genes defined as elevated in the brain downloaded on 2017-11-22 from https://www.proteinatlas.org/search/tissue_specificity_rna%3Acerebral+cortex%3Belevated+AND+sort_by%3Atissue+specific+score?format=tsv. These genesets have been used for to screen for aberrations with potential functional relevance in genes associated with monogenic disease or cancer or with a high expression in brain (Table 1 main text). Also, “refFlat_bed” contains the data from "refFlat.sorted-ucsc-hg19.bed" used for annotation of RefSeq genes in CNVs.

SUPPLEMENTAL METHODS

Proband cohort and ethics approval

PD patients were diagnosed by board-examined movement disorder specialists according to consensus criteria of the German Society of Neurology, which are similar to the UK PD Society Brain Bank criteria for diagnosis of PD[10]. PD patients were tested for known PD-causing genetic mutations using a multiplex PCR based Ion AmpliSeq Custom Panel (200bp design; Thermo Fisher Scientific, Waltham, USA). Pathogenic mutations in the coding regions of the genes *SNCA* (*163890), *PARK2* (*602544), *UCHL1* (*191342), *PINK1* (*608309), *DJ1* (*602533), *LRRK2* (*609007), *ATP13A2* (*610513), *GIGYF2* (*612003), *HTRA2* (*606441), *PLA2G6* (*603604), *FBXO7* (*605648), *VPS35* (*601501), *EIF4G1* (*600495), *TBP* (*600075), *MAPT* (*157140), *HLA-DRA* (*142860), and *RIT2* (*609592) were excluded. In patient "VK2" we identified a heterozygous frameshift mutation in the *FBXO7* gene (Parkinsons disease 15, autosomal recessive), and in patient "RJO" we revealed a heterozygous deletion of exons 3-5 in the *PARK2* gene (Parkinsons disease 2, autosomal recessive). In both patients, we were not able to detect a further variant on the second allele. Patients with complicated hereditary spastic paraplegia were included into the study following positive genetic testing for compound heterozygous mutations in *SPG11* (transcript: NM_025137.3; "4AA": c.3036C>A p.(Tyr1012*) and c.5798delC p.(Ala1933Valfs*18); "K22": c.267G>A p.(Trp89*) and c.1457-2A>G p.Glu486fs508*; "G7G": c.3075dupA p.(Glu1026Argfs*4) and c.6204A>G p.(Val2075Aspfs*18))[11,12]. Patients with monogenic intellectual disability were included into the study following positive genetic testing using Illumina TruSight One Sequencing Panel on a MiSeq System or whole exome sequencing on a Illumina HiSeq2000 system (Illumina, San Diego, CA, USA), which resulted in the identification of likely pathogenic variants in *SCN2A*. Study approval was granted by the local ethics committees (No. 4485, FAU Erlangen-Nuernberg, Germany; and No StV I 1/09 Canton of Zurich) and all participants gave written informed consent prior to inclusion into the study.

Generation of iPSC and NPC

Human iPSC were reprogrammed using retroviral transduction of the transcriptions factors *POU5F1* (OCT3/4), *SOX2* (SOX2), *KLF4* (KLF4) and *MYC* (c-MYC) as previously described.[13] For non-integrating Sendai reprogramming (SiPSC) with

Yamanaka transcription factors, fibroblasts were infected with CytoTuneTM-iPS Sendai Reprogramming Kit (Thermo Fisher Scientific, Waltham, USA) and processed according to the manufacturer's protocol. The underlying Institutional Review Board approval (Nr. 4120: “Generierung von humanen neuronalen Modellen bei neurodegenerativen Erkrankungen”) and informed consent are available at the movement disorder clinic at the Department of Molecular Neurology, Universitätsklinikum Erlangen (Erlangen, Germany). All the human iPSC lines were screened for pluripotency and for stable karyotype using G-banding chromosomal analysis (Figure S1; File S2). Two human iPSC clones from each PD patient and age- and sex-matched controls (File S1) were differentiated into NPCs as previously described[14]. In brief, human iPSC were detached five to seven days after passaging using collagenase IV (Gibco; Thermo Fisher Scientific, Waltham, USA) treatment for 20 min at 37°C, 5% CO2. Cell colonies were resuspended in human embryonic stem cells (hESC) medium (80% KO-DMEM, 20% KO serum replacement, 1% non-essential amino acids, 1% Penicillin/Streptavidin (all from Thermo Fisher Scientific, Waltham, USA), 1mM ß-Mercaptoethanol (Sigma-Aldrich, St. Louis, USA) supplemented with the small molecules 1 µM LDN, 10 µM SB, 3 µM Chir, 0.5 µM Purmorphoamine (PMA, all from Tocris, Bristol, UK)) and cultured on ultra-low adhesion plates. After two days of incubation, the medium was changed to N2B27 medium (50% DMEM/F12, 50% Neurobasal Medium, 1:200 N2, 1:100 B27 (all from Thermo Fisher Scientific, Waltham, USA)) supplemented with the same small molecules. On day four, the medium was changed to smNPC medium (N2B27 medium supplemented with 3 µM Chir, 0.5 µM PMA and 150 µM Ascorbic acid (AA; Sigma-Aldrich, St. Louis, USA)). After two more days of suspension culture, cell colonies were replated on geltrex-coated (Gibco; Thermo Fisher Scientific, Waltham, USA) 12-well plates in smNPC medium supplemented with Rho kinase inhibitor Y27532 (RI, Tocris, Bristol, UK). The medium was changed every other day and cells were passaged once a week as single cells in a ratio of 1:6 to 1:9. After at least five passages, NPCs were differentiated into DA neurons.

Pluripotency Testing using Immunocytochemistry and Fluorescence activated cell sorting (FACS)

iPSCs and NPCs were fixed with 4% paraformaldehyde (Merck, Darmstadt, Germany) and blocked/permeabilized using 3% donkey serum (PAN Biotech,

Aidenbach, Germany) and 0.1% TritonX-100 in PBS (both Sigma, St. Louis, USA). A combination of primary antibodies (Nanog - catalogue number: AF1997, host: goat, diluted 1:200 (R&D Systems, Minneapolis, USA); Nestin - catalogue number: MAB5326, host: mouse, diluted 1:300 (Millipore, Burlington, USA); Oct3/4 - catalogue number: sc5279, host: mouse (Santa Cruz, Santa Cruz, USA); Sox2 - catalogue number: 3579S, host: rabbit (Cell signaling, Danvers, USA)) were incubated at 4°C overnight before incubation of fluorescent-labeled secondary antibodies (all Dianova, Hamburg, Germany) at room temperature for 1h followed by counterstaining with DAPI (1:10,000 in PBS). Images were acquired on an AxioObserver equipped with an ApoTome using Zen blue software (Carl Zeiss, Oberkochen, Germany).

To determine the percentage of TRA-1-60-positive cells iPSCs were detached using accutase (Sigma, St. Louis, USA). For each line 100,000 – 300,000 cells were used per staining. After washing with PBS twice cells were incubated with Alexa Fluor 488 anti-human TRA-1-60-R antibody or the Alexa Fluor 488 Mouse IgM, κ Isotype Ctrl antibody (both Biolegend, San Diego, USA) for 10min at room temperature. FACS was performed using a FACSCalibur (Becton, Dickinson and Company, Franklin Lakes, USA) and FlowJo software (FlowJo LLC, Ashland, USA).

Conventional karyotyping and FISH

All samples were tested by conventional karyotyping with a resolution > 10 Mb. Standard protocols were used for fibroblast preparation. For iPSC preparation, standard protocols for lymphocyte cultures were used. About 20% of the samples (44.0% of the fibroblasts, 15.2% of the iPSCs) analyzed by conventional karyotyping at the Institute of Human Genetics, Friedrich-Alexander-Universität Erlangen-Nürnberg (FAU) showed structural or numerical aberrations, partially as mosaicism, and were excluded from further studies (File S2). Note that for some fibroblasts and iPSC lines conventional karyotyping had been performed at the respective center and the data is not collected centralized. All unremarkable cultures were expanded to receive sufficient material for following experiments.

Fluorescence in-situ hybridization (FISH) was performed on metaphase spreads from cultivated iPSCs of the SiPSC clone “i82A-S1-004” with the 17q sub-telomeric probe Pac 17q362-k4 according to standard protocols.

DNA purification and genetic fingerprinting

Genomic DNA was extracted according to standard procedures using the Qiagen DNeasy Blood & Tissue Kit (Qiagen, Venlo, Netherlands). Genetic fingerprinting of most cultures was performed by multiplex PCR with 20 polymorphic markers located on 15 different chromosomes (PowerPlexTM21; Promega, Fitchburg, USA) and analyzed on an automatic capillary sequencer (ABI3500dx; Thermo Fisher Scientific, Waltham, USA) to validate their identity and to exclude handling errors during the numerous culturing steps. For the samples without PowerPlex analysis the sample identity was confirmed from CMA data (File S2).

Molecular karyotyping

Molecular karyotyping was performed with an Affymetrix CytoScan HD array (Affymetrix, Santa Clara, USA), in accordance with the supplier's instructions. Copy number data of arrays fulfilling the quality criteria (MAPD ≤ 0.25, Waviness SD ≤ 0.12, and SNPQC ≥15) were analyzed using the Affymetrix Chromosome Analysis Suite 3.1.0.15 (ChAS; Affymetrix, Santa Clara, USA) software with regards to aberrations minimally sizing 100 kb. The CMA data (log2 ratio of the intensities and allele peak distribution) for each analyzed culture was visually compared to the respective fibroblast by a trained expert (M.K.). To exclude germline variants, we screened for aberrations ≥100 kilobases (kb) absent from the parental line using 3,500 in-house samples and the Database of Genomic Variants (DGV) as controls[15] as well as in-house control samples to identify benign CNVs. All specifications confer to February 2009 Human Genome Assembly (hg19) and Human Genome Build 37, respectively.

Fingerprinting and ancestry estimation from CMA data

SNP genotypes were extracted from the CMA ".cyhd.cychp" files using an in-house script to convert the HDF5 file format to plain text. These genotypes where then converted into a multi-sample VCF file using PLINK.[16] This VCF file was then used as input for the akt-toolkit version dfe6dd0[3] to calculate pairwise sample kinship and ancestry for all samples with CMA data.

Exome sequencing and alignment

For the exome analysis a total of 34 samples from different samples of four individuals were selected. For each of these four individuals a blood sample, a fibroblast sample, two independent RiPSC samples, one or two SiPSC samples and up to two NPC sample were available. For two individuals higher passage (P30, P40) RiPSC samples were included. Enrichment and library preparation for exome sequencing was performed on DNA using the SureSelect Human All Exon V6 kit (Agilent Technologies, Santa Clara, USA). Sequencing was carried out on three machine runs with 125 bp paired-end reads on an Illumina Hiseq 2500 system (Illumina, Inc., San Diego, USA). After demultiplexing, quality and adapter trimming were performed on the reads using BBduk from the BBmap/BBTools package v37.17. Read alignment to the hg19 reference genome from the GATK[17] (Genome Analysis Toolkit) bundle was performed with BWA-MEM[18] version 0.7.15. Resulting SAM files were converted to BAM files and sorted with sambamba[19] version 0.6.6. Duplicate reads were marked with samblaster[20] version 0.1.24. As in-house controls 53 individuals with germline exome sequencing from the same machine runs were selected and BAM files were prepared with the same protocol as for the study samples. See File S1 for detailed sample and control descriptions.

Variant calling and annotation of exome data

Concurrent calling of somatic variants for the 34 study samples and 53 in-house controls was performed on the final BAM files using freebayes[4] v1.1.0. To allow sensitive detection of potential somatic variants with a lower allele-fraction the parameter “min-alternate-count” was set to 3 and the parameter “min-alternate-fraction” to 0.05 based on previous experiences with somatic variant calling in cancer. As an additional somatic variant calling strategy we performed pairwise calling of all cell samples against their respective blood sample and a normal panel of 53 in-house control samples using MuTect2[5] from GATK version 3.7-0 with standard parameters. The UnifiedGenotyper (UG) from GATK version 3.7-0 was used for concurrent germline variant calling on all samples, which was then used as input for fingerprinting analysis using the akt-toolkit (see below). All resulting variant files were normalized by left aligning indels and splitting multiallelic sites using bcftools[6] version 1.2. For annotation of the resulting variant files, SnpEff/ SnpSift[21,22] were used with dbNSFP[23] version 2.9.3 and variant frequencies from the gnomAD database version

2.0.1, COSMIC[24] database version 81 and ClinVar[25] database version 20171002 using the files provided from the respective website.

Variant filtering for exome data

Potential somatic variant positions from the freebayes calls were filtered to have a read coverage of ≥10 in the blood sample (BLS) and in the respective cell culture sample (CCS), an alternative allele fraction (AF) in the BLS of ≤1%, AF in the CCS sample of ≥5%, allelic depth for the alt allele in the CCS of ≥5 and the sum of the allelic depth for the alt allele in the 53 in-house controls being ≤2x the allelic depth for the alt allele in the CCS. For the MuTect2 call set variants had to additionally pass the internal algorithm filter ("PASS"). Only coding and splice region variant positions not reported more than 30 times in the gnomAD database or having at least 10 observations in the COSMIC database were considered for both call sets. Such filtered variants with an AF in the CCS of ≥30% were considered to be present in most cells from the respective culture and classified as fixed somatic variants ("fix") while variants with an AF <30% were classified as present in a subclonal part of cells only and classified as low frequency ("low"). Variants in genomic regions difficult to access by short read sequencing were excluded using DangerTrack[26] and the genomic regions of the five genes (*SOX2* chr3[hg19]:181427712-181434223, *POU5F1*: chr6[hg19]:31130114-31140451, *POU5F1B*: chr8[hg19]:128425857-128431441, *MYC*: chr8[hg19]:128746315-128755680, *KLF4*: chr9[hg19]:110245133-110254047) corresponding to the transcription factors used for reprogramming were also excluded to avoid false positive calls. Subsequently, the resulting lists were examined using the IGV browser.[27]

Somatic CNV calling from exome aligned read files

Somatic copy number variation (CNV) calling from exome data was performed using CNVkit[28] version 0.9.2 with standard parameters against the 53 in-house control samples (File S3; Figure S6).

Telomere content analysis from exome data

Telomerehunter[8] version 1.0.4 and telomerecat[7] version 3.1.2 were used to estimate telomere length from the sequenced of target telomeric reads in the exome sequencing BAM files of all study samples and in-house control samples (File S5; Figure S5).

Quality control of exome data

Quality control on final BAM files was performed using qualimap[29] version 2.2. See File S2 for detailed coverage statistics.

Mitochondrial dosage analysis from exome data

To calculate the relative mitochondrial genome ratio, the average coverage of the mitochondrial genome (chrM) was normalized to the on-target coverage of the chromosome 1 (chr1) both computed by qualimap[29].

Fingerprinting and ancestry estimation from exome data

Sample kinship and ancestry was confirmed by pairwise comparison of the germline UG variant calls using the akt-toolkit version dfe6dd0[3].

Contamination analysis of exome data

ContEst[9] from the GATK was used to estimate the degree of cross-sample contamination in the exome files. The BBSketch MinHash algorithm from BBTools in the sendsketch-script implementation was used to screen for evidence of cell culture contamination with microorganisms in the exome files. See File S2 for detailed quality control results.

Plotting of figures and statistical analysis

All plotting for figures and statistical analyses were performed in R version 3.4.3 with the RStudio IDE (RStudio, Inc.) using the data provided as supplementary Excel (Microsoft Corporation, Redmond, USA) files. Libraries used were: ggplot2, tidyverse, Gviz, trackViewer, plyr, readr, readxl, Rmisc, ggsignif, ggrepel, cowplot, svglite, ggbio, scales, GenomicRanges. Individual Figures were additionally composed using Illustrator / Photoshop CC 2018 (both: Adobe Systems, San José, USA) or Inkscape 0.92.2 (https://inkscape.org/) if the figure could not be directly composed in R.

LINKS

BBmap/BBTools: https://jgi.doe.gov/data-and-tools/bbtools/

freebayes: https://github.com/ekg/freebayes/

bcftools: https://samtools.github.io/bcftools/bcftools.html

gnomAD: http://gnomad.broadinstitute.org/

COSMIC: http://cancer.sanger.ac.uk/cosmic/

ClinVar: https://www.ncbi.nlm.nih.gov/clinvar/

akt: https://github.com/Illumina/akt/

DangerTrack: https://github.com/DCGenomics/DangerTrack/

telomerecat: https://pypi.python.org/pypi/telomerecat/

telomerehunter: https://pypi.python.org/pypi/telomerehunter/

DGV: http://dgv.tcag.ca/

RStudio: https://www.rstudio.com/

Inkscape: https://inkscape.org/

SUPPLEMENTAL REFERENCES

1 Seltmann, S. *et al.* hPSCreg--the human pluripotent stem cell registry. *Nucleic Acids Res* **44**, D757-763, doi:10.1093/nar/gkv963 (2016).

2 Genomes Project, C. *et al.* A global reference for human genetic variation. *Nature* **526**, 68-74, doi:10.1038/nature15393 (2015).

3 Arthur, R., Schulz-Trieglaff, O., Cox, A. J. & O'Connell, J. AKT: ancestry and kinship toolkit. *Bioinformatics* **33**, 142-144, doi:10.1093/bioinformatics/btw576 (2017).

4 Garrison, E. & Marth, G. Haplotype-based variant detection from short-read sequencing. *arXiv preprint arXiv:1207.3907* (2012).

5 Cibulskis, K. *et al.* Sensitive detection of somatic point mutations in impure and heterogeneous cancer samples. *Nature biotechnology* **31**, 213-219, doi:10.1038/nbt.2514 (2013).

6 Li, H. A statistical framework for SNP calling, mutation discovery, association mapping and population genetical parameter estimation from sequencing data. *Bioinformatics* **27**, 2987-2993, doi:10.1093/bioinformatics/btr509 (2011).

7 Farmery, J. H. R., Smith, M. L., Diseases, N. B.-R. & Lynch, A. G. Telomerecat: A ploidy-agnostic method for estimating telomere length from whole genome sequencing data. *Sci Rep* **8**, 1300, doi:10.1038/s41598-017-14403-y (2018).

8 Feuerbach, L. *et al.* TelomereHunter: telomere content estimation and characterization from whole genome sequencing data. *bioRxiv* (2016).

9 Cibulskis, K. *et al.* ContEst: estimating cross-contamination of human samples in next-generation sequencing data. *Bioinformatics* **27**, 2601-2602, doi:10.1093/bioinformatics/btr446 (2011).

10 Hughes, A. J., Daniel, S. E., Kilford, L. & Lees, A. J. Accuracy of clinical diagnosis of idiopathic Parkinson's disease: a clinico-pathological study of 100 cases. *J Neurol Neurosurg Psychiatry* **55**, 181-184 (1992).

11 Hehr, U. *et al.* Long-term course and mutational spectrum of spatacsin-linked spastic paraplegia. *Ann Neurol* **62**, 656-665, doi:10.1002/ana.21310 (2007).

12 Schneider-Gold, C. *et al.* Monozygotic twins with a new compound heterozygous SPG11 mutation and different disease expression. *J Neurol Sci* **381**, 265-268, doi:10.1016/j.jns.2017.09.005 (2017).

13 Havlicek, S. *et al.* Gene dosage-dependent rescue of HSP neurite defects in SPG4 patients' neurons. *Hum Mol Genet* **23**, 2527-2541, doi:10.1093/hmg/ddt644 (2014).

14 Reinhardt, P. *et al.* Derivation and expansion using only small molecules of human neural progenitors for neurodegenerative disease modeling. *PLoS One* **8**, e59252, doi:10.1371/journal.pone.0059252 (2013).

15 Iafrate, A. J. *et al.* Detection of large-scale variation in the human genome. *Nat Genet* **36**, 949-951, doi:10.1038/ng1416 (2004).

16 Purcell, S. *et al.* PLINK: a tool set for whole-genome association and population-based linkage analyses. *Am J Hum Genet* **81**, 559-575, doi:10.1086/519795 (2007).

17 McKenna, A. *et al.* The Genome Analysis Toolkit: a MapReduce framework for analyzing next-generation DNA sequencing data. *Genome research* **20**, 1297-1303, doi:10.1101/gr.107524.110 (2010).

18 Li, H. Aligning sequence reads, clone sequences and assembly contigs with BWA-MEM. *arXiv preprint arXiv:1303.3997* (2013).

19 Tarasov, A., Vilella, A. J., Cuppen, E. & Nijman, I. J. Sambamba: fast processing of NGS alignment formats. … (2015).

20 Faust, G. G. & Hall, I. M. SAMBLASTER: fast duplicate marking and structural variant read extraction. *Bioinformatics* **30**, 2503-2505 (2014).

21 Cingolani, P., Patel, V. M., Coon, M. & Nguyen, T. Using Drosophila melanogaster as a model for genotoxic chemical mutational studies with a new program, SnpSift. *Frontiers in …* (2012).

22 Cingolani, P., Platts, A., Wang, L. L., Coon, M. & Nguyen, T. A program for annotating and predicting the effects of single nucleotide polymorphisms, SnpEff: SNPs in the genome of Drosophila melanogaster strain …. *Fly*, doi:10.4161/fly.19695 (2012).

23 Liu, X., Jian, X. & Boerwinkle, E. dbNSFP v2. 0: a database of human non-synonymous SNVs and their functional predictions and annotations. *Human mutation*, doi:10.1002/humu.22376 (2013).

24 Forbes, S. A., Beare, D. & Gunasekaran, P. COSMIC: exploring the world's knowledge of somatic mutations in human cancer. *Nucleic acids …* (2014).

25 Landrum, M. J. *et al.* ClinVar: public archive of relationships among sequence variation and human phenotype. *Nucleic Acids Res* **42**, D980-985, doi:10.1093/nar/gkt1113 (2014).

26 Dolgalev, I., Sedlazeck, F. & Busby, B. DangerTrack: A scoring system to detect difficult-to-assess regions. *F1000Research* **6**, 443, doi:10.12688/f1000research.11254.1 (2017).

27 Robinson, J. T. *et al.* Integrative genomics viewer. *Nature biotechnology* **29**, 24-26, doi:10.1038/nbt.1754 (2011).

28 Talevich, E., Shain, A. H. & Botton, T. CNVkit: genome-wide copy number detection and visualization from targeted DNA sequencing. *PLoS computational …*, doi:10.1371/journal.pcbi.1004873 (2016).

29 Garcia-Alcalde, F. *et al.* Qualimap: evaluating next-generation sequencing alignment data. *Bioinformatics* **28**, 2678-2679, doi:10.1093/bioinformatics/bts503 (2012).
